# Designing and implementing an experimental survey on knowledge and perceptions about alcohol warning labels

**DOI:** 10.1002/mpr.2016

**Published:** 2024-05-17

**Authors:** Daniela Correia, Alexander Tran, Daša Kokole, Maria Neufeld, Aleksandra Olsen, Tiina Likki, Carina Ferreira‐Borges, Jürgen Rehm

**Affiliations:** ^1^ WHO Regional Office for Europe Copenhagen Denmark; ^2^ EPIUnit – Institute of Public Health University of Porto Porto Portugal; ^3^ Institute for Mental Health Policy Research Centre for Addiction and Mental Health Toronto Ontario Canada; ^4^ Department of Health Promotion CAPHRI Care and Public Health Research Institute Maastricht University Maastricht the Netherlands; ^5^ Campbell Family Mental Health Research Institute Centre for Addiction and Mental Health Toronto Ontario Canada; ^6^ Department of Psychiatry University of Toronto Toronto Ontario Canada; ^7^ Dalla Lana School of Public Health University of Toronto Toronto Ontario Canada; ^8^ Center for Interdisciplinary Addiction Research (ZIS) Department of Psychiatry and Psychotherapy University Medical Center Hamburg‐Eppendorf (UKE) Hamburg Germany; ^9^ Faculty of Medicine Institute of Medical Science University of Toronto Toronto Ontario Canada; ^10^ Program on Substance Abuse Public Health Agency of Catalonia Program on Substance Abuse & WHO CC Public Health Agency of Catalonia Barcelona Spain

**Keywords:** alcohol, approximating population distributions, experimental design, health warning labels, web‐based survey

## Abstract

**Objectives:**

This paper describes the design and implementation of an online survey experiment to investigate the effects of alcohol warning labels on alcohol‐related knowledge, risk perceptions and intentions.

**Method:**

The survey collected self‐reported data from 14 European countries through two waves of data collection with different recruitment strategies: dissemination via social media and public health agencies was followed by paid‐for Facebook ads. The latter strategy was adopted to achieve broader population representation. Post‐stratification weighting was used to match the sample to population demographics.

**Results:**

The survey received over 34,000 visits and resulted in a sample size of 19,601 participants with complete data on key sociodemographic characteristics. The responses in the first wave were over‐representing females and higher educated people, thus the dissemination was complemented by the paid‐for Facebook ads targeting more diverse populations but had higher attrition rate.

**Conclusion:**

Experiments can be integrated into general population surveys. Pan‐European results can be achieved with limited resources and a combination of sampling methods to compensate for different biases, and statistical adjustments.

## 1 INTRODUCTION

Alcohol is a major risk factor for the burden of disease, causing about 3 million deaths and more than 130 million DALYs lost worldwide in 2016 (K. Shield et al., 2020). The European Union is the region with the highest level of alcohol consumption with seven out of the 10 countries with the highest alcohol consumption being member states (WHO Global Health Observatory, 2019). To reduce alcohol‐related harm, different policy options have been proposed, with pricing policies, availability and marketing restrictions usually considered as most effective and cost‐effective (Babor et al., 2022; Chisholm et al., 2018).

Labels on alcohol products, including health warnings, are one of the policy options endorsed by the World Health Organization (WHO) (WHO European Region, 2022b; WHO's Alcohol Drugs and Addictive Behaviours Unit, 2022) with scarcer evidence base compared to tobacco, where health warnings have been established as an important part of a comprehensive policy strategy (Flor et al., 2021). The majority of the existing studies experimentally examining the impact of labels on different variables have been done in English‐speaking countries (Kokole et al., 2021), and despite a few country‐specific studies focusing on particular populations in mainland Europe (Annunziata et al., 2019; Glock & Krolak‐Schwerdt, 2013; Lacoste‐Badie et al., 2022; Morgenstern et al., 2021; Staub et al., 2022), there remains a notable shortage of comprehensive studies on alcohol labelling in this region (Jané‐Llopis et al., 2020).

In 2022, the WHO Regional Office for Europe launched the Evidence into Action Alcohol Project (EVID‐ACTION) (WHO European Region, 2022c), with one of its key objectives being the development of the evidence base to support the implementation of effective alcohol health warnings. As a first step, an online experiment was designed to provide insight into the impact of different health messages on participants' knowledge of alcohol‐related harms, their risk perception and drinking intentions, as well as on the participants' perceptions of the different labels. A research protocol has been developed and uploaded in Open Science Framework (OSF) before the start of the study (WHO European Region, 2022a).

We opted to employ a web‐based study to efficiently conduct a randomized experiment on the effect of different alcohol labels across multiple countries, in multiple languages. However, as we also wanted to determine knowledge and risk perceptions on a population level, we needed to ensure a sufficient quota for populations who are usually underrepresented in web‐based convenience samples such as the elderly or less‐educated groups (Wyatt, 2000). Such convenience sampling methods, although not statistically representative, have data that is comparable to probability survey methods (Barratt et al., 2017). Furthermore, true representative samples are extremely difficult if not impossible to obtain given the high rate of non‐response and the incomplete sampling frames (Rehm, Kilian, Rovira, et al., 2021).

Thus, this paper describes the procedures employed for data collection and weighting needed to achieve a final sample that can approximate the population characteristics in the 14 participating countries. Its use can result in a efficient approach for multi‐country research.

## 2 METHOD

### 2.1 Aim and development of the survey

With the objective of investigating impact of different health messages on participants' knowledge of alcohol‐related harms, their risk perception and drinking intentions, as well as on the participants' perceptions of the different labels, an online survey with an experimental component was conducted via WHO's online platform Data‐Form (LimeSurvey) (Schmitz, 2015), covering 14 countries (Austria, Belgium, Estonia, France, Germany, Ireland, Latvia, Lithuania, Netherlands, Norway, Portugal, Slovenia, Spain, and Sweden). The questionnaire was originally designed and developed in English and was translated into 12 languages (Catalan, German, Dutch/Flemish, French, Estonian, Latvian, Lithuanian, Norwegian, Portuguese, Slovenian, Spanish, and Swedish) by country counterparts from national institutions and the translations were double checked by the native speakers in the research team or on the national level. Based on an a priori power analysis of effects and planned statistical analyses for country interactions as described in the protocol (WHO European Region, 2022a), we aimed to reach a minimum sample size of 384 participants per country. Moreover, 1050 participants per country would be necessary to enable country‐specific analyses for some of the variables.

The survey was a mixed‐effects study, with label conditions measuring a between‐subjects effect, and pre‐post knowledge as well as other variables measuring within‐subjects effects. The experiment consisted of six different randomized label conditions: one control with no message, and five experimental labels with different written messages and/or images displayed on the front label of an alcoholic beverage container. Of the five experimental labels, three included a message specifically linking alcohol consumption to cancer risk. The details regarding labels' attributes can be found in Table A1.

The measures used in the pre‐ and post‐manipulation included knowledge of health conditions related to alcohol including cancer, perceived general health risk, perceived cancer risk due to alcohol consumption, and intention to reduce alcohol consumption. Post‐manipulation‐only measures included self‐reported attention to label and items evaluating respondents' perceptions of different labels. Demographic information (age, gender and educational attainment level as a measure of socio‐economic status), AUDIT‐C (Babor et al., 2001) and perceived social norms regarding alcohol consumption were also assessed as covariate and/or potential moderator variables. The order of presenting survey questions was fixed across all questionnaire versions, ensuring consistency among participants. However, to minimize order effects, the order of multiple‐choice answers within specific questions was randomized. The complete questionnaire can be found in the Supporting Information S1.

The selection of countries aimed to include the three main drinking cultures present in the European Union (Grant, 2013; Popova et al., 2007), as well as variations within these cultures. Portugal, France, Slovenia, and Spain follow the Mediterranean style, characterized by regular alcohol consumption, particularly wine with meals, and cultural disapproval of public drunkenness. However, some generational changes have been observed in Spain, shifting from wine to beer preference among younger individuals, and higher acceptance of public drunkenness (Llamosas‐Falcón & Rehm, 2023). The Central and Western European pattern is predominantly associated with beer‐drinking countries, where beer consumption is frequent, with or without meals. This pattern is typical of Germany but also observed in Austria, Belgium, the Netherlands, and Ireland. The Northern European pattern revolves around spirits consumption, characterized by non‐daily drinking, sporadic binge drinking episodes, and a higher tolerance for public drunkenness. Norway and Sweden fall into this pattern but have recently undergone a change towards wine drinking. This group also contains the Baltic countries of Estonia, Latvia, and Lithuania, that show historically higher levels of alcohol consumption due to their affiliation with the former Soviet Union (Rehm, Štelemėkas, et al., 2021).

The inclusion criteria for the study were individuals aged 18 years old or older that reported consuming alcohol within the past 12 months, that is, current drinkers. Despite the legal drinking age in some countries such as Germany and Belgium being 16, individuals under 18 years old would require parental consent for participating and thus that was set for defining the exclusion criteria across all countries. The only exception was Lithuania, where the legal age of consent is 20 years old, and so the survey distributed in Lithuania was changed to exclude participants younger than 20 years of age.

Participants were volunteers (i.e., were not compensated for completing the survey) that followed a survey link to an online experiment, which could be completed through their smartphone or tablet/computer. To maintain the full anonymity of participants, no IP addresses were collected or retained, and participants provided basic demographic information (gender, estimated household income, age within 5‐year age categories, country, and type of place of residence). All participants provided informed consent before participating in the study and exemption on the EU level was provided by the Data Protection Office in Departament de Salut, Generalitat de Catalunya (Barcelona, Spain, DPD #21/2022). Ethical clearance was granted by the WHO Collaborating Centre for Addiction and Mental Health in Toronto, Canada (Centre for Addiction and Mental Health, Research Ethics Board, #095/2022).

### 2.2 Recruitment strategies for data collection

The survey respondents were composed of a non‐probabilistic convenience sample that was collected in 14 countries in two separate but consecutive survey waves, with different dissemination methods. Despite not aiming to recruit a truly representative sample, efforts were made to reach a wide range of populational groups by age group, sex, and educational attainment level, as these factors are known to be associated with alcohol consumption (T. F. Babor et al., 2022).

For the first sample wave, national partners were responsible for dissemination in their respective countries. The survey was distributed in each country's native language(s), but language selection was available at the beginning of the online questionnaire. Participants were recruited via multiple online channels, disseminated as widely as possible using a snowball effect through WHO collaborating centres, Ministries of Health in respective countries, and non‐governmental organizations (NGOs) with members in several of the participating countries. It included mailing lists, official organization‐based social media accounts, government websites, and other general survey contact lists. Detailed information on the dissemination channels used for each country, date of dissemination and languages in which the survey was available can be found in Table A2, in the Supporting Information S1.

Preliminary descriptive analyses of the data collected in the first wave indicated that the sample was composed mostly of females and people with tertiary education. As a consequence, some population subgroups (e.g., males with high school education or lower) were only represented by a small number of individuals per country in the sample, which compromised the ability to draw conclusions based on the data at the country level. Given the skewed demographic representation of respondents, interpretations of the perceptions of the different labels in different European countries, one of the primary objectives of the survey, would suffer from this major limitation. Thus, a second sample wave was conducted on Meta Verse Platforms, namely using paid‐for Facebook ads, for the same set of countries as in the first wave. It aimed to complement the first sample wave by targeting people who had lower educational attainment levels (less than tertiary education), with the goal set as 2000 clicks per country over 2 weeks, for a total budget of 14,000 euros (1000 euros per country). To better manage the budget available, all countries started the campaigns with a daily budget of 50 euros, aiming for an average of 143 daily clicks, and further adjustments were made depending on the evolution of cost per click (CPC). Most countries successfully reached the goal with the initial daily budget of 50 euros, but five (Austria, Estonia, the Netherlands, Sweden, and Norway) needed multiple campaigns with budget adjustments to reach the goal. The detailed results per country of paid‐for Facebook ads are shown in Table 1. The overall cost per click was 0.28 euros, and the overall completion rate was around 30%, with the lowest rate observed in Lithuania (9.1%) and the highest in Germany (62.8%). Collapsing the completion rate and cost per click yielded a measure of effectiveness defined as cost per completed response, which ranged between 0.33 and 3.01 euros and overall was 0.93 euros/completed response.

**TABLE 1:** Dissemination results of paid‐for Facebook ad, by country.

Country	Budget spent (EUR)	Number of clicks	Overall CPC (EUR)	Completion rate	Cost per completed response (EUR)
Austria	831.80	2017	0.41	48.0%	0.86
Belgium	714.69	2135	0.33	27.5%	1.22
Estonia	820.85	2040	0.40	31.7%	1.27
France	610.42	3249	0.19	38.2%	0.49
Germany	602.85	2886	0.21	62.8%	0.33
Ireland	703.94	2096	0.34	27.8%	1.21
Latvia	615.76	2785	0.22	33.8%	0.65
Lithuania	694.99	2547	0.27	9.1%	3.01
Netherlands	753.81	2020	0.37	35.4%	1.05
Norway	1381.26	2146	0.64	24.2%	2.66
Portugal	583.91	5578	0.10	13.9%	0.75
Slovenia	629.56	2592	0.24	33.6%	0.72
Spain	600.93	4284	0.14	27.4%	0.51
Sweden	1135.17	2179	0.52	21.0%	2.48
Total	10,679.94	38,554	0.28	29.9%	0.93

Abbreviation: CPC, cost per click.

### 2.3 Post‐stratification weighting

Given that this project aimed to understand and compare country‐level differences on various measures, including perceptions of labels with different messages on alcoholic beverages and baseline levels of knowledge for the risk of cancer due to alcohol consumption, we aimed to match the sample to population‐level statistics for each country. In doing so, we would reduce the amount of bias due to a country being of a specific demographic, and thus obtain more accurate estimations of descriptive statistics at the country level. In addition, when studying causal effects, it can improve the estimation of regression models' coefficients by correcting for heteroskedasticity or being used to identify average partial effects in the presence of unmodeled heterogeneity of effects (Solon et al., 2015).

For the sample from each country, the number of respondents across our key sociodemographic variables was compared to the actual population distribution from EUROSTAT (Eurostat, 2022). Standardized post‐stratification adjustment weights were computed to approximate the sample to the EUROSTAT population distribution by sex (female or male), age group (18–34, 35–54, 55 or above) and educational attainment level (non‐tertiary education or tertiary education), for a sub‐sample with complete data on these key sociodemographic characteristics. The choice to group ages into 18–34, 35–54, and 55 or above aligns with EUROSTAT's 5‐year intervals and helps distinguish between young, middle‐aged, and older adults. This simplifies our data for compatibility and allows us to explore distinct alcohol‐related behaviours across these age categories, as they are known to differ in their drinking habits (T. F. Babor et al., 2022). Weights were calculated by country, meaning that the sum of the weights by country equals its sample size.

During the weighting process, high weights, reaching up to 19, were assigned to certain groups to address sample underrepresentation. This assignment, crucial for achieving post‐stratification goals, introduced the potential for undue influence from observations exhibiting potential outlier behaviour. To mitigate this, a decision was made to trim weights exceeding 5, and subsequently, the distribution of weights by country was standardized again. While this specific cut‐off lacked universal prescription, it represented a pragmatic choice to balance accurate post‐stratification weighting with outlier prevention—aligned with practical considerations in survey research (Korn & Graubard, 1999). This chosen cut‐off ensured result stability without compromising the representativeness of the sample. Weights exceeding 5 were trimmed for 24 out of the 168 sociodemographic groups used in the weighing procedure.

A significance level of 5% was assumed as well as independence between observations. All analyses were performed using R software version 4.2.1 for windows.

## 3 RESULTS

By the end of the data collection period (May 24, 2023) the survey link had been visited by more than 34,000 participants. Of those, around two‐thirds (63.2%, *n* = 20,183) provided informed consent, met the minimum age and drinking status criteria for participation, and reached the end of the survey. The attrition rate was lower in the first sample wave as compared to the second sample wave (34.0% vs. 45.5%). In addition, participants with a country of residence outside of the 14 countries considered for this study were excluded (*n* = 202), resulting in a final sample size of *n* = 19,981 participants. Of those, 19,601 provided complete information on sex (female or male) and educational attainment level. Around 23% of the responses were completed through smartphone, and the remaining by tablet/computer.

**FIGURE 1:**
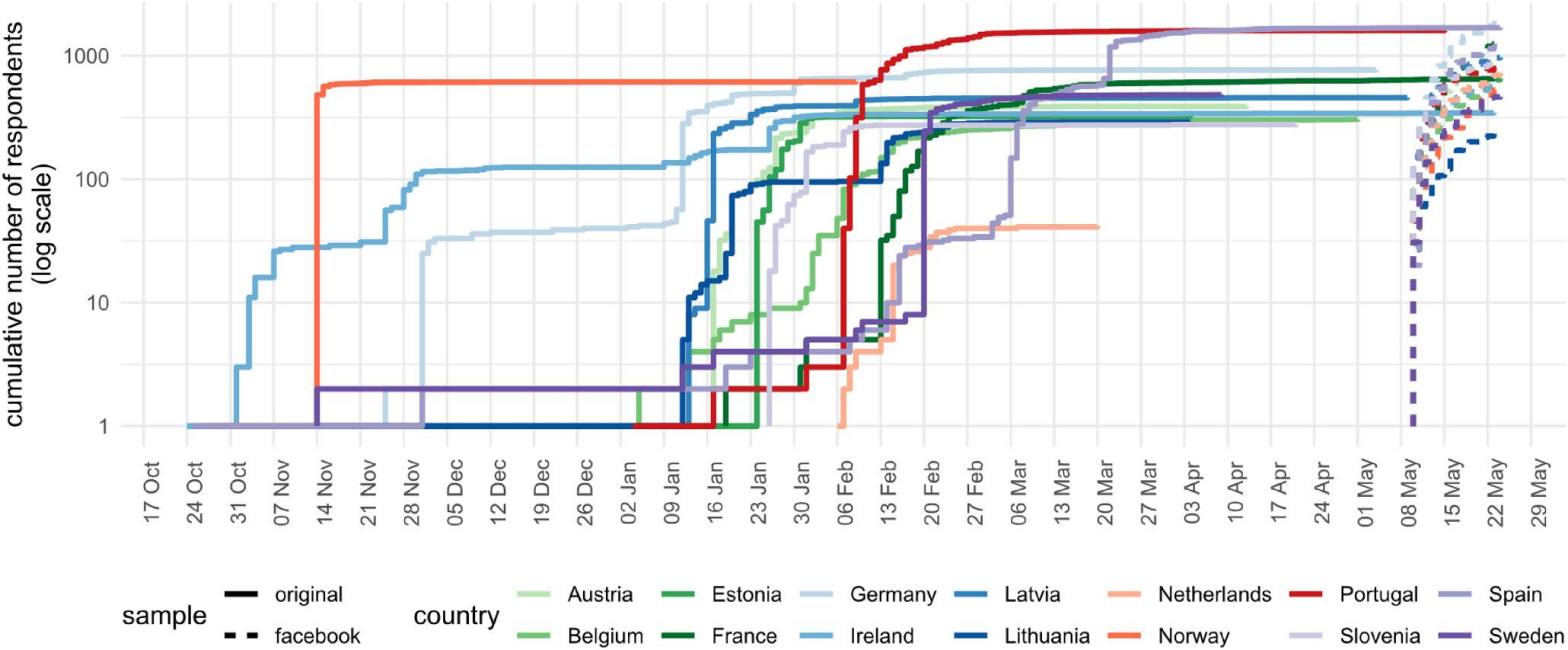
Cumulative number of valid submissions throughout data collection, by country of residence and sample wave, between October 2022 and May 2023.

The distribution of the final sample by sample wave and sociodemographic characteristics can be found in Table 2, and the evolution of the cumulative number of respondents is plotted in Figure 1. The first sample wave began collection on October 24, 2022 for four countries, and three weeks later, on November 14th, for three others. The data collection for the remaining countries started between the fourth of January and the sixth of February 2023, and increases in the number of respondents were observed until the end of March 2023. Most countries surpassed the 300 participants in the first sample wave, except for Lithuania (*n* = 291), Slovenia (*n* = 277) and the Netherlands (*n* = 42).

**TABLE 2:** Survey sample characteristics by the wave of data collection.

Characteristic	Overall *N* = 19,601	1st wave *N* = 8081 (41.2%)^a^	2nd wave (facebook) *N* = 11,520 (58.8%)^a^
Country			
Austria	1356	387 (28.5%)	969 (71.5%)
Belgium	885	298 (33.7%)	587 (66.3%)
Estonia	969	323 (33.3%)	646 (66.7%)
France	1876	636 (33.9%)	1240 (66.1%)
Germany	2565	754 (29.4%)	1811 (70.6%)
Ireland	923	340 (36.8%)	583 (63.2%)
Latvia	1386	444 (32.0%)	942 (68.0%)
Lithuania	511	280 (54.8%)	231 (45.2%)
Netherlands	758	42 (5.5%)	716 (94.5%)
Norway	1126	607 (53.9%)	519 (46.1%)
Portugal	2345	1570 (67.0%)	775 (33.0%)
Slovenia	1144	274 (24.0%)	870 (76.0%)
Spain	2826	1652 (58.5%)	1174 (41.5%)
Sweden	931	474 (50.9%)	457 (49.1%)
Age group			
18–34 years old	11,851	3024 (25.5%)	8827 (74.5%)
35–54 years old	5026	3533 (70.3%)	1493 (29.7%)
55+ years old	2724	1524 (55.9%)	1200 (44.1%)
Sex			
female	10,007	5306 (53.0%)	4701 (47.0%)
male	9594	2775 (28.9%)	6819 (71.1%)
Education level			
secondary or less	9123	1662 (18.2%)	7461 (81.8%)
tertiary	10,478	6419 (61.3%)	4059 (38.7%)

^a^ *n* (%).

The second sample wave was collected for all countries simultaneously, during a 2‐week period between the 10th and May 24, 2023. It represents 58.8% of the total sample size, comprising between 40% and 70% of the final sample in most countries. The exceptions were Portugal, for which it represents only a third of the sample size (33.2%), and the Netherlands, where the paid‐for Facebook ads sample represents the great majority of the data collected (94.5%). As for absolute values, the second sample wave collected more than 400 participants for most countries, only with the exception of Lithuania (*n* = 238), due to the low completion rate (9.1%).

### 3.1 Comparison between sample waves

As previously mentioned, the second wave of data collection aimed to complement the first wave by targeting specific populational groups that were under‐represented when compared to country statistics, due to the dissemination methods applied in the first wave. As shown in Table 2, the original sample consisted mostly of people with tertiary education (79.4%), representing between 67.1% and 96.8% of the sample at the country level. Despite not aiming for sample representativeness at the country level, the high percentage of people with tertiary education in the sample implied a bias in responses and a lack of population groups with lower socioeconomic status, which would compromise further statistical analyses and the generalizability of the study conclusions. The majority of the sample is also composed of women (65.7%), which was observed for all countries with the exception of Germany (33.5%) and included more people in younger age groups (37.4% were 18–34 years old, 43.7% were 35–54 years old, and 18.9% were aged 55 or older).

Thus, paid‐for, targeted Facebook ads were necessary, specifically directed at people with lower educational attainment of all age groups and sex. It resulted in a sizeable change in the distribution of the sample proportions by education level, as expected, with almost two‐thirds (64.8%) of the respondents collected during the second wave having secondary education or lower. The same effect was observed by sex, with males representing most of the respondents (59.2%) of the second sample wave, despite not being directly targeted by the paid‐for Facebook ads. On the other hand, as a consequence of using social media as a tool for dissemination, this sample presents a higher proportion of younger respondents (76.9% were 18–34 years old, 12.9% were 35–54 years old, and 10.3% were aged 55 or older).

### 3.2 Coverage of the population by the sample data

Table 3 presents the sample size of each country according to the three key sociodemographic characteristics (i.e., sex, age and educational attainment level), and compares unweighted and weighted sample distribution with the country's respective distributions in 2022, obtained from EUROSTAT (Eurostat, 2022). The number of observations in each group is higher than 100 for all countries, falling behind only for adults aged 55 or older in Estonia (*n* = 46) and Slovenia (*n* = 48), and middle‐aged adults (35–54 years old) in the Netherlands (*n* = 87). Before weighting, and as compared to the EUROSTAT characteristics, survey samples had a higher proportion of adults under 35 years old and a lower proportion of the remaining age groups for all countries except for Sweden. Unweighted education marginal distributions were successfully approximated in Austria, Estonia, and Germany. As for sex, the unweighted samples were never successful approximations of EUROSTAT distributions.

**TABLE 3:** Sample distribution of socioeconomic characteristics by country of residence and comparison with country distribution according to EUROSTAT.

	Sample			Population EUROSTAT (2022) %
	*N*	Unweighted % (95% CI)	Weighted % (95% CI)	
Austria *N* = 1356				
Age groups (%)				
18–34	825	60.8 (58.2, 63.4)	28.4 (26.0, 30.9)	28.1
35–54	366	27.0 (24.7, 29.5)	38.3 (35.0, 41.8)	38.0
55+	165	12.2 (10.5, 14.1)	33.3 (29.4, 37.4)	33.9
Sex (%)				
Female	580	42.8 (40.1, 45.5)	49.9 (46.3, 53.6)	50.4
Male	776	57.2 (54.5, 59.9)	50.1 (46.4, 53.7)	49.6
Educational level (%)				
Secondary or less	939	69.2 (66.7, 71.7)	67.3 (64.1, 70.3)	67.6
Tertiary	417	30.8 (28.3, 33.3)	32.7 (29.7, 35.9)	32.4
Belgium *N* = 885				
Age groups (%)				
18–34	582	65.8 (62.5, 68.9)	29.7 (26.6, 33.1)	29.6
35–54	173	19.5 (17.0, 22.3)	36.8 (32.0, 41.9)	36.9
55+	130	14.7 (12.5, 17.2)	33.4 (28.6, 38.6)	33.5
Sex (%)				
Female	377	42.6 (39.3, 45.9)	50.0 (45.2, 54.8)	50.2
Male	508	57.4 (54.1, 60.7)	50.0 (45.2, 54.8)	49.8
Educational level (%)				
Secondary or less	424	47.9 (44.6, 51.3)	59.4 (55.0, 63.7)	59.6
Tertiary	461	52.1 (48.7, 55.4)	40.6 (36.3, 45.0)	40.4
Estonia *N* = 969				
Age groups (%)				
18–34	710	73.3 (70.3, 76.0)	30.8 (27.2, 34.6)	27.2
35–54	213	22.0 (19.4, 24.8)	44.3 (38.8, 49.9)	39.2
55+	46	4.7 (3.53, 6.33)	25.0 (19.6, 31.4)	33.5
Sex (%)				
Female	659	68.0 (65.0, 70.9)	53.7 (48.1, 59.1)	51.4
Male	310	32.0 (29.1, 35.0)	46.3 (40.9, 51.9)	48.6
Educational level (%)				
Secondary or less	601	62.0 (58.9, 65.1)	56.8 (51.5, 62.0)	61.8
Tertiary	368	38.0 (34.9, 41.1)	43.2 (38.0, 48.5)	38.2
France *N* = 1876				
Age groups (%)				
18–34	1235	65.8 (63.6, 68.0)	29.7 (27.3, 32.3)	28.0
35–54	379	20.2 (18.4, 22.1)	38.9 (35.3, 42.7)	37.0
55+	262	14.0 (12.4, 15.6)	31.3 (28.0, 34.9)	35.0
Sex (%)				
Female	782	41.7 (39.4, 44.0)	48.6 (45.0, 52.1)	51.6
Male	1094	58.3 (56.0, 60.6)	51.4 (47.9, 55.0)	48.4
Educational level (%)				
Secondary or less	588	31.3 (29.3, 33.5)	61.1 (58.0, 64.0)	63.3
Tertiary	1288	68.7 (66.5, 70.7)	38.9 (36.0, 42.0)	36.7
Germany *N* = 2565				
Age groups (%)				
18–34	1857	72.4 (70.6, 74.1)	29.0 (27.0, 31.0)	27.3
35–54	468	18.2 (16.8, 19.8)	37.8 (34.9, 40.8)	35.6
55+	240	9.4 (8.27, 10.6)	33.2 (30.1, 36.6)	37.1
Sex (%)				
Female	721	28.1 (26.4, 29.9)	47.0 (43.9, 50.1)	50.0
Male	1844	71.9 (70.1, 73.6)	53.0 (49.9, 56.1)	50.0
Educational level (%)				
Secondary or less	1851	72.2 (70.4, 73.9)	69.1 (66.3, 71.7)	70.9
Tertiary	714	27.8 (26.1, 29.6)	30.9 (28.3, 33.7)	29.1
Ireland *N* = 923				
Age groups (%)				
18–34	531	57.5 (54.3, 60.7)	30.3 (27.2, 33.5)	29.9
35–54	242	26.2 (23.4, 29.2)	42.2 (37.8, 46.8)	41.7
55+	150	16.3 (14.0, 18.8)	27.5 (23.1, 32.4)	28.5
Sex (%)				
Female	515	55.8 (52.5, 59.0)	50.2 (45.7, 54.6)	50.8
Male	408	44.2 (41.0, 47.5)	49.8 (45.4, 54.3)	49.2
Educational level (%)				
Secondary or less	379	41.1 (37.9, 44.3)	53.3 (48.9, 57.5)	53.9
Tertiary	544	58.9 (55.7, 62.1)	46.7 (42.5, 51.1)	46.1
Latvia *N* = 1386				
Age groups (%)				
18–34	896	64.6 (62.1, 67.2)	29.4 (26.7, 32.3)	25.8
35–54	341	24.6 (22.4, 27.0)	44.2 (40.0, 48.5)	38.8
55+	149	10.8 (9.19, 12.5)	26.4 (22.4, 30.9)	35.4
Sex (%)				
Female	957	69.0 (66.5, 71.5)	50.3 (46.1, 54.4)	52.9
Male	429	31.0 (28.5, 33.5)	49.7 (45.6, 53.9)	47.1
Educational level (%)				
Secondary or less	673	48.6 (45.9, 51.2)	60.3 (56.6, 63.8)	65.1
Tertiary	713	51.4 (48.8, 54.1)	39.7 (36.2, 43.4)	34.9
Lithuania *N* = 511				
Age groups (%)				
18–34	240	47.0 (42.6, 51.4)	31.2 (26.1, 36.9)	26.7
35–54	167	32.7 (28.7, 37.0)	39.3 (32.7, 46.3)	37.7
55+	104	20.4 (17.0, 24.2)	29.5 (23.0, 37.0)	35.6
Sex (%)				
Female	410	80.2 (76.5, 83.5)	52.4 (45.4, 59.3)	52.5
Male	101	19.8 (16.5, 23.5)	47.6 (40.7, 54.6)	47.5
Educational level (%)				
Secondary or less	107	20.9 (17.5, 24.8)	51.6 (44.8, 58.3)	58.7
Tertiary	404	79.1 (75.2, 82.5)	48.4 (41.7, 55.2)	41.3
Netherlands *N* = 758				
Age groups (%)				
18–34	514	67.8 (64.3, 71.1)	30.7 (27.3, 34.4)	30.5
35–54	87	11.5 (9.34, 14.0)	34.6 (29.3, 40.3)	35.1
55+	157	20.7 (17.9, 23.8)	34.6 (29.8, 39.7)	34.4
Sex (%)				
Female	349	46.0 (42.5, 49.7)	49.7 (44.6, 54.8)	50.0
Male	409	54.0 (50.3, 57.5)	50.3 (45.2, 55.4)	50.0
Educational level (%)				
Secondary or less	435	57.4 (53.8, 60.9)	61.1 (56.2, 65.8)	61.4
Tertiary	323	42.6 (39.1, 46.2)	38.9 (34.2, 43.8)	38.6
Norway *N* = 1126				
Age groups (%)				
18–34	518	46.0 (43.1, 49.0)	31.2 (28.5, 34.1)	31.2
35–54	360	32.0 (29.3, 34.8)	37.6 (34.4, 40.8)	37.6
55+	248	22.0 (19.7, 24.6)	31.2 (28.0, 34.5)	31.2
Sex (%)				
Female	664	59.0 (56.0, 61.9)	49.3 (46.1, 52.6)	49.3
Male	462	41.0 (38.1, 44.0)	50.7 (47.4, 53.9)	50.7
Educational level (%)				
Secondary or less	620	55.1 (52.1, 58.0)	58.2 (55.1, 61.3)	58.2
Tertiary	506	44.9 (42.0, 47.9)	41.8 (38.7, 44.9)	41.8
Portugal *N* = 2345				
Age groups (%)				
18–34	1060	45.2 (43.2, 47.2)	27.4 (25.4, 29.5)	25.4
35–54	916	39.1 (37.1, 41.1)	42.2 (39.2, 45.2)	39.1
55+	369	15.7 (14.3, 17.3)	30.4 (27.2, 33.8)	35.6
Sex (%)				
Female	1295	55.2 (53.2, 57.2)	48.6 (45.5, 51.7)	52.4
Male	1050	44.8 (42.8, 46.8)	51.4 (48.3, 54.5)	47.6
Educational level (%)				
Secondary or less	897	38.3 (36.3, 40.3)	70.3 (68.2, 72.3)	72.5
Tertiary	1448	61.7 (59.7, 63.7)	29.7 (27.7, 31.8)	27.5
Slovenia *N* = 1144				
Age groups (%)				
18–34	899	78.6 (76.1, 80.9)	28.5 (25.5, 31.6)	24.2
35–54	197	17.2 (15.1, 19.6)	46.8 (41.9, 51.8)	39.8
55+	48	4.2 (3.14, 5.57)	24.7 (19.6, 30.6)	36.0
Sex (%)				
Female	643	56.2 (53.3, 59.1)	48.5 (43.6, 53.5)	48.7
Male	501	43.8 (40.9, 46.7)	51.5 (46.5, 56.4)	51.3
Educational level (%)				
Secondary or less	673	58.8 (55.9, 61.7)	63.2 (58.4, 67.7)	65.9
Tertiary	471	41.2 (38.3, 44.1)	36.8 (32.3, 41.6)	34.1
Spain *N* = 2826				
Age groups (%)				
18–34	1675	59.3 (57.4, 61.1)	29.0 (26.8, 31.3)	25.0
35–54	760	26.9 (25.3, 28.6)	44.0 (40.8, 47.2)	41.7
55+	391	13.8 (12.6, 15.2)	27.1 (24.0, 30.3)	33.3
Sex (%)				
Female	1501	53.1 (51.3, 55.0)	46.6 (43.5, 49.7)	50.6
Male	1325	46.9 (45.0, 48.7)	53.4 (50.3, 56.5)	49.4
Educational level (%)				
Secondary or less	504	17.8 (16.4, 19.3)	57.9 (55.1, 60.7)	63.7
Tertiary	2322	82.2 (80.7, 83.6)	42.1 (39.3, 44.9)	36.3
Sweden *N* = 931				
Age groups (%)				
18–34	309	33.2 (30.2, 36.3)	30.8 (27.9, 33.9)	30.8
35–54	357	38.3 (35.2, 41.6)	36.9 (33.6, 40.3)	36.9
55+	265	28.5 (25.6, 31.5)	32.3 (29.0, 35.7)	32.3
Sex (%)				
Female	554	59.5 (56.3, 62.7)	49.1 (45.7, 52.6)	49.1
Male	377	40.5 (37.3, 43.7)	50.9 (47.4, 54.3)	50.9
Educational level (%)				
Secondary or less	432	46.4 (43.2, 49.7)	58.0 (54.6, 61.3)	58.0
Tertiary	499	53.6 (50.3, 56.8)	42.0 (38.7, 45.4)	42.0

**FIGURE 2:**
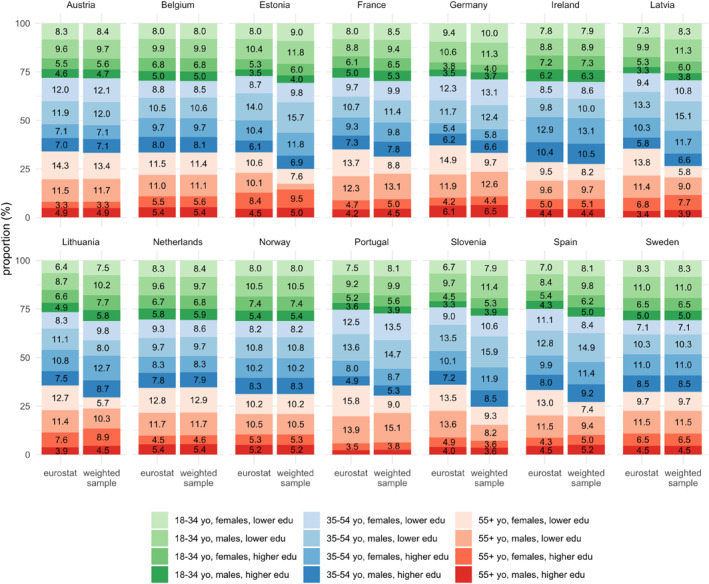
Comparison of cross‐distribution of age group, sex and educational attainment between the weighted sample and the country according to EUROSTAT. Labels representing percentages lower than 3% were omitted.

After weighing, age, sex, and education, marginal distributions matched the countries' actual distribution in most cases. Austria, Belgium, Ireland, the Netherlands, Norway, and Sweden were perfectly matched in all marginal distributions; the remaining countries failed to match at least one group (e.g., the weighted proportion of adults aged 55 years old or older in Germany was estimated as 33.2%, 95%CI: 30.1%–36.6%, whereas EUROSTAT indicates that it represents 37.1% in the population). The average absolute deviation from EUROSTAT values was 2.2 percentual points, with the highest deviations observed for the older age group in Slovenia (weighted sample: 24.7%, 95%CI = 19.6–30.6%; EUROSTAT: 36.0%) and in Latvia (weighted sample: 26.4%, 95%CI = 22.4–30.9%; EUROSTAT: 35.4%). The comparison of the key socioeconomic characteristic's joint distribution between the weighted sample and country is represented in Figure 2, while comparison of marginal distribution can be found in Figure A1. Moreover, unweighted, and weighted distribution of participants by label condition can be found in Figure A2. Adjustments made through weighting did not change the distribution, resulting in balanced sample sized before and after weighting.

## 4 DISCUSSION

This paper provides a comprehensive description of the design of an experimental survey and the dissemination strategies employed to collect a convenience sample across 14 countries. The implementation of the two strategies yielded distinct results in terms of response rates, sample size but complementary sample characteristics. The differences observed underscore the importance of thoughtful selection and execution of dissemination methods, as they directly impact the composition and characteristics of the obtained sample.

The initial wave of data collection revealed the presence of a biased sample compared to the distribution of sex, education and age in the general population of the respective countries, primarily influenced by the distribution channels employed. It was observed that most channels used for this sample wave predominantly attracted participants with higher levels of education, indicating the need to avoid relying solely on these specific channels in future studies. Additionally, the effectiveness of dissemination efforts varied depending on the reach of national counterparts, resulting in notable discrepancies in sample sizes across countries. Notably, despite having a high number of respondents, and relatively high statistical power, large groups of the general population were missing, as highlighted by previous research, underscoring the importance and value of investigating sample distributions, and employing weighting procedures (Kilian et al., 2021). Close monitoring of responses during data collection is essential to identify underrepresented sub‐populations and make necessary adjustments.

Targeted sampling methods have the potential to approximate the sample to its population characteristics. Paid‐for Facebook ads emerge as a viable approach to recruit survey participants due to their ease of implementation, efficient management by small teams within short timeframes, and relatively low costs compared to traditional survey methods (Kapp et al., 2013; Schneider & Harknett, 2022). However, targets might not be fully achieved, and the efficacy of paid‐for Facebook ads in recruiting targeted participants varied across countries. In our study, the average cost per valid response was 0.93 EUR, with variations ranging from 3.01 to 0.33 EUR. Other studies have reported higher costs, such as 2.18 CAD (roughly 1.51 EUR at the time of this study) (Shaver et al., 2019). These cost discrepancies point out the need to consider country‐specific factors when determining the feasibility and cost‐effectiveness of recruitment strategies.

The observed heterogeneity between samples highlights the potential bias introduced by self‐selected, non‐probabilistic surveys. Such surveys often necessitate the application of statistical adjustments, such as post‐stratification weighting, to mitigate biases (Greenacre, 2016; Mäkelä, 2021; Rehm, Kilian, & Manthey, 2021; Rehm, Kilian, Rovira, et al., 2021; Wright, 2005), as implemented in our study. However, it resulted in relatively high weights for some particular sub‐populations, due to the inherent heterogeneity of the sample. Hence, weight trimming was required, resulting in small deviations when comparing the cross‐sectional sample distributions of key socioeconomic characteristics with population data, at the country level. Moreover, multi‐country surveys can result in massive samples, which might compromise the most traditional statistical analysis as they are overpowered for testing many hypotheses (Case & Ambrosius, 2007). It is important to exercise caution and consider the practical significance and effect size in addition to statistical significance when interpreting results from large samples (Sullivan & Feinn, 2012).

The study has some limitations: first, we cannot claim a representative survey, as such a characteristic depends on probabilistic sampling strategies, and inclusive sampling frame and no response biases present drinking (Kruskal & Mosteller, 1979). Such representative surveys do not seem to be possible in alcohol studies in the EU. First, most probabilistic sampling frames based on households exclude key groups of heavy alcohol consumers, that is the homeless and some institutionalised populations (K. D. Shield & Rehm, 2012). Second, high non‐response bias usually leads to the underestimation of alcohol use, as evidence in the discrepancy between level of consumption estimated via surveys and via sales in a country (Rehm et al., 2007). Thus, the presented solution is an attempt to produce the best possible results with limited resources given the current possibilities (Rehm, Kilian, Rovira, et al., 2021). Finally, it needs to be mentioned that for the hypothesis testing of the experimental part, representativeness is not necessary (Rothman et al., 2013). Another limitation is that our sample is based on drinkers and was weighted against the general population. While we cannot exclude a bias here, as abstainers may have different sociodemographic characteristics than alcohol consumers, such a bias will be small, given the only a relatively small minority in the EU abstains from alcohol (WHO European Region, 2019).

In conclusion, Pan‐European results can be achieved with limited resources when adopting a combination of different sampling techniques to achieve an overall quota sampling based on joint distributions of key characteristics, complemented by necessary statistical weighting adjustments. It is imperative, however, that such surveys are designed and implemented following best practice guidelines to maximize response rates, such as keeping the survey length short and making it available for several devices. In addition, integrating experiments into surveys is a practice that is not yet commonly employed but should be embraced more frequently, as it offers a valuable means of obtaining causal inferences.

## AUTHOR CONTRIBUTIONS

**Daniela Correia**: Formal analysis; writing ‐ original draft; investigation; data curation; methodology; validation; visualization. **Alexander Tran**: Methodology; software; conceptualization; investigation; data curation. **Daša Kokole**: Conceptualization; methodology; investigation; visualization. **Maria Neufeld**: Conceptualization; funding acquisition; resources; methodology; project administration. **Aleksandra Olsen**: Project administration; resources. **Tiina Likki**: Conceptualization; resources; project administration. **Carina Ferreira‐Borges**: Supervision; funding acquisition; resources; project administration. **Jürgen Rehm**: Supervision; project administration; writing ‐ review & editing; visualization; validation; methodology.

## CONFLICT OF INTEREST STATEMENT

None. Carina Ferreira‐Borges, Tiina Likki, Maria Neufeld and Aleksandra Olsen are staff members of the World Health Organization; Daniela Correia, Daša Kokole, Alexander Tran, and Jürgen Rehm are WHO consultants. The authors alone are responsible for the views expressed here and these do not necessarily represent the decisions or the stated policy of WHO.

## ETHICS STATEMENT

Ethical clearance was granted by the WHO Collaborating Centre for Addiction and Mental Health in Toronto, Canada (Centre for Addiction and Mental Health, Research Ethics Board, #095/2022).

## CONSENT

Participants provided informed consent before participating in the study and exemption on the EU level was provided by the Data Protection Office in Departament de Salut, Generalitat de Catalunya (Barcelona, Spain, DPD #21/2022).

## PERMISSION TO REPRODUCE MATERIAL FROM OTHER SOURCES

Permission is not required.

## CLINICAL TRIAL REGISTRATION

None.

## Supporting information

Supporting Information S1

## Data Availability

The data that support the findings of this study are available from the corresponding author, [D.C.], upon reasonable request.
